# Funding for rare tumor research in China under discussion

**DOI:** 10.1093/nsr/nwab106

**Published:** 2021-06-18

**Authors:** Weijie Zhao

**Affiliations:** NSR news editor based, Beijing

‘Throughout the world, medical research has never been equitable. Diseases with more patients and better publicity always grasp more attention, get more funding, and are better studied,’ said Wenming Wu, a digestive surgeon and the vice-President of the Peking Union Medical College Hospital.

The Chinese government released the first List of Rare Diseases in 2018, and the National Rare Disease Diagnosis and Treatment Cooperation Network of 324 hospitals was established in 2019. Additionally, the Chinese Ministry of Science and Technology invested 38 million yuan (∼6 million US dollars) in the National Rare Diseases Registry System of China, which included 186 rare disease cohorts with 65 418 registered patients by 30 May 2021. The government is also adjusting its medical insurance policies to better support patients with rare diseases and their families.

However, the 2018 list of rare diseases does not include rare tumors. ‘Rare tumor’ is not a definition but a concept, defined as tumors with annual incidence less than 6/100 000 (EU) or 15/100 000 (US). Differing incidence rates between countries mean that their lists of rare tumors vary greatly, for example, esophageal squamous cell carcinoma is a rare disease in the United States, but a common cancer in China. ‘Since the large population of China, Chinese researchers use a lower incidence definition of 2.5/100 000 for clinical research, but it still covers 520 000 new patients each year, accounting for 13.3% of all cancer patients,’ said Ning Li, Director of China's National Cancer Center Office.

On 27–28 May, the National Natural Science Foundation of China (NSFC) held a special Forum on rare tumors. Clinicians and scientists working on rare tumors gathered to discuss potential research programs for NSFC. ‘We have to compete with common tumors such as lung and liver cancers. It's extremely difficult for rare tumor research programs to get funded,’ said Jie Chen, a physician at the First Affiliated Hospital of Sun Yat-sen University whose specialty is neuroendocrine tumors. Moreover, besides the ‘rare tumors’ in the general sense, rare molecular subtypes of common tumors are also poorly studied.

The Surveillance of Rare Cancers in Europe identified as many as 186 rare cancers. Research funding from NSFC is unlikely to cover all rare cancers. ‘Funding should go for tumors with relatively high incidence in the Chinese population, mucosal melanoma is an example,’ said Jun Guo, vice-Dean of the Beijing Cancer Hospital. Melanoma incidence in China is much lower than that of western countries. However, about 96% of melanoma cases in the western counties are chronic sun-damaged (CSD) melanoma or non-CSD melanoma (5-year survival rates >90%). But in China, more than 70% melanoma cases are acral melanoma or mucosal melanoma, with 5-year survival rates around 54% and 30%, respectively.

On the other hand, as China's national funding agency for basic research, NSFC may be more interested in the proposals relating to fundamental scientific problems. ‘Rare tumor research may enrich our understanding of common tumors. For example, neuroendocrine tumor may be a good model to study cancer metastasis,’ said George Fu Gao, vice-President of NSFC and Director-General of the Chinese Center for Disease Control and Prevention.

‘Different rare tumors may share common molecular targets. It may be a good idea to search for common targets and potential common therapies,’ proposed Hongtao Yu, Dean of the School of Life Sciences of Westlake University. According to Yu, a number of biomedical technologies could be used in rare tumor research, including organoids and other disease models, multiomics analyses, high-throughput drug screening, as well as artificial intelligence.

This NSFC Forum on rare tumors is one of the Shuangqing Forums. Every year, NSFC holds about 30 Shuangqing Forums on different topics. However, being selected as a topic does not guarantee more funding from NSFC; this depends on further evaluations.

Throughout the world, patients with rare tumors are disadvantaged by poor scientific understanding of these diseases. They often need much more time to be diagnosed, have very few applicable drugs and therapies, and bear heavy economic burdens. To improve their quality of life and to form a better medical care system for them, strong coherent supports are needed not only from NSFC, but also the Ministry of Science and Technology, National Health Commission, and other funding agencies.

